# Using a Tablet-Based App to Deliver Evidence-Based Practices for Suicidal Patients in the Emergency Department: Pilot Randomized Controlled Trial

**DOI:** 10.2196/23022

**Published:** 2021-03-01

**Authors:** Linda A Dimeff, David A Jobes, Kelly Koerner, Nadia Kako, Topher Jerome, Angela Kelley-Brimer, Edwin D Boudreaux, Blair Beadnell, Paul Goering, Suzanne Witterholt, Gabrielle Melin, Vicki Samike, Kathryn M Schak

**Affiliations:** 1 Evidence Based Practice Institute, Inc Seattle, WA United States; 2 The Catholic University of America Washington, DC United States; 3 Department of Emergency Medicine, Psychiatry, and Population and Quantitative Health Sciences University of Massachusetts Medical School Worcester, MA United States; 4 Evaluation Specialists Carlsbad, WA United States; 5 Mental Health and Addiction Allina Health Minneapolis, MN United States; 6 Department of Psychiatry and Psychology Mayo Clinic Rochester, MN United States

**Keywords:** suicide, emergency department, digital technology, suicide prevention

## Abstract

**Background:**

Emergency departments (EDs) have the potential to provide evidence-based practices for suicide prevention to patients who are acutely suicidal. However, few EDs have adequate time and personnel resources to deliver recommended evidence-based assessment and interventions. To raise the clinical standard of care for patients who are suicidal and seeking psychiatric crisis services in the ED, we developed Jaspr Health, a tablet-based app for direct use by such patients, which enables the delivery of 4 evidence-based practices.

**Objective:**

This study aims to evaluate the feasibility, acceptability, and effectiveness of Jaspr Health among suicidal adults in EDs.

**Methods:**

Patients who were acutely suicidal and seeking psychiatric crisis services participated in an unblinded pilot randomized controlled trial while in the ED. Participants were randomly assigned to Jaspr Health (n=14) or care as usual (control; n=17) groups. Participants were assessed at baseline, and a 2-hour posttest using self-report measures and a semistructured interview were conducted.

**Results:**

Conditions differed significantly at baseline with regard to age but not other demographic variables or baseline measures. On average, participants had been in the ED for 17 hours before enrolling in the study. Over their lifetime, 84% (26/31) of the sample had made a suicide attempt (mean 3.4, SD 6.4) and 61% (19/31) had engaged in nonsuicidal self-injurious behaviors, with an average rate of 8.8 times in the past 3 months. All established feasibility and acceptability criteria were met: no adverse events occurred, participants’ app use was high, Jaspr Health app user satisfaction ratings were high, and all participants using Jaspr Health recommended its use for other suicidal ED patients. Comparisons between study conditions provide preliminary support for the effectiveness of the app: participants using Jaspr Health reported a statistically significant increase in receiving 4 evidence-based suicide prevention interventions and overall satisfaction ratings with their ED experience. In addition, significant decreases in distress and agitation, along with significant increases in learning to cope more effectively with current and future suicidal thoughts, were observed among participants using Jaspr Health compared with those receiving care as usual.

**Conclusions:**

Even with limited statistical power, the results showed that Jaspr Health is feasible, acceptable, and clinically effective for use by ED patients who are acutely suicidal and seeking ED-based psychiatric crisis services.

**Trial Registration:**

ClinicalTrials.gov NCT03584386; https://clinicaltrials.gov/ct2/show/NCT03584386

## Introduction

### Background

With 48,344 suicides reported in 2018 (1 every 11 min and 132 per day) [1], suicide remains the 10th leading cause of death across all age groups [2] and the second leading cause of death among people aged 10-44 years [3] in the United States. Moreover, suicide rates have significantly increased over the past two decades, making suicide one of the few health outcomes proving difficult to impact [2,4]. Specifically, the annual suicide rate increased by 33% between 1999 and 2017 [5], from 10.5 to 14.8 per 100,000. From 2009 to 2018, the rate increased from 19.23 to 22.79 per 100,000 for males and from 4.88 to 6.18 per 100,000 for females [6]. Nearly 16 million people worldwide make a suicide attempt on an annual basis [7,8], with approximately 1.4 million adults in the United States making attempts in 2018 [7,9]. In addition, a staggering 12 million American adults thought seriously about trying to kill themselves [10]. Although death by firearms remains the most common method of suicide in the United States, intentional self-poisoning with substances, including opioids, accounts for more than 5000 suicides annually [11].

These staggering increases have led to soaring numbers of emergency department (ED) visits for suicide attempts and ideation in recent years [12]. Approximately 575,000 people are treated annually in US EDs for injuries because of self-harm [13], and 1% of all ED visits involve suicidal ideation [12]. Between 2006 and 2013, ED visits for suicidal ideation increased by 12% annually. During this same period, costs of ED visits because of suicidal ideation increased by over 20% annually—from US $600 million to US $2.2 billion.

Suicidal patients pose special and difficult challenges for EDs [14]. On average, these patients wait for care more than 3 times longer than those with medical emergencies [14,15], which leads to the problem of *boarding*, where the patient is waiting (and still under observational status) in the ED for an inpatient or residential bed to become available [16-18]. Factors contributing to extended wait times include a lack of available inpatient beds (72% of patients who are suicidal are referred to inpatient hospitalization) [12] and inadequate access to hospital-based mental health providers to provide suicide risk assessments, stabilize crises, and help safely transition patients home [19]. Boarding then leads to crowding, poor patient experience, lower quality care [20], delays in treatment, and morbidity and mortality [21] as beds that might otherwise be used to treat patients with life-threatening medical conditions [19] are used for patients who are suicidal while they await treatment or transfer to an inpatient unit or a residential facility. Suicide crises also have a tremendous financial impact on EDs. One study found that every behavioral health ED visit prevented 2.2 beds from turning over, costing EDs an average of US $2264 in lost revenue per visit [15].

### ED-Based Interventions to Reduce Suicidal Deaths

EDs can also play a consequential role in reducing suicides by providing evidence-based care for a population at high risk for suicide [22]. For example, studies have shown that up to 25% of patients who seek ED services following a suicide attempt will make another attempt and up to 10% will die by suicide. Of those who do, a substantial proportion will have visited the ED for suicidality the year before they die [23-25]. ED-based interventions thus offer a unique opportunity [26] to intervene for this high-risk population where delivery of evidence-based interventions in the ED could reduce annual deaths from suicide by as much as 20% [27]. For these reasons, a number of public health policy initiatives have recommended increased delivery of suicide prevention efforts during ED visits [28]. Accordingly, in recent years, The Joint Commission has required improved screening for suicidality. By July 2021, The Joint Commission will also require EDs to create suicide crisis safety plans for all patients who are acutely suicidal [29]. The reality, however, is that few EDs have the time and personnel resources to perform these best practices [30].

The use of electronic tablets in health care by providers and patients has exponentially increased over the past decade because of their portability, efficiency, and range of functionality. For patients who are suicidal in the ED, a tablet encased in a strong protective case could easily be brought to the patient’s bedside, play psychoeducational videos, and allow users to generate text content to complete a comprehensive, evidence-based suicide risk assessment. Such an app could replace hours of unstructured waiting that characterizes the typical ED experience with robust suicide prevention interventions.

### Study Purpose

We sought to create a digital technology for use in EDs to increase the delivery of suicide prevention evidence-based practices without adding to personnel needs in an effort to reduce suicidal behavior with an ultimate goal of saving lives while also improving the quality of ED care delivered to those who are suicidal. Jaspr Health was developed over a span of several years using an iterative process of development and best practices in user-centered design [31-34]. Extensive feedback was sought from ED patients who were suicidal (n=89) and their ED-based care team (n=105) from 4 large health care systems across the United States. At its core is the Collaborative Assessment and Management of Suicidality (CAMS), a highly adaptable evidence-based suicide prevention intervention developed by Jobes [35], for use by clinicians in engaging, assessing, and treating patients who are suicidal. CAMS uses a chart-ready documentation tool, the Suicide Status Form, to serve as a clinical roadmap guiding assessment, treatment planning, and ongoing tracking of risk and care disposition. Developed two decades earlier, CAMS contains many recommended practices, including a comprehensive suicide risk assessment, stabilization planning, and lethal means safety counseling [36]. It has been used as a brief intervention, an add-on to an existing treatment, and a short-term suicide-focused treatment. Beyond suicide risk factors and warning signs, CAMS identifies and treats patient-articulated *drivers* of suicide, as defined as those problems and other issues that compel a person to consider suicide, for example, trauma, romantic breakup, or financial issues. To date, 5 published randomized controlled trials (RCTs) support CAMS efficacy as a clinical suicide prevention intervention [37-41]. CAMS was integrated into Jaspr Health.

We conducted this preliminary RCT in EDs to examine the feasibility, acceptability, and effectiveness of Jaspr Health for adults who were acutely suicidal in the ED. We sought to determine if patients in the midst of a profound suicide crisis would be able and willing to use a tablet-based app to complete a comprehensive suicide risk assessment, build a crisis stability plan, undergo lethal means counseling, and learn behavioral skills to improve their capacity to tolerate future crises. Would patients feel as if using an app diminished or compromised their overall ED satisfaction and experience? In addition, would providers and health care systems allow their patients who are suicidal to interact with a tablet? And would Jaspr Health produce outcomes that might justify its continued use—in the ED and other care pathways? Results from this pilot can assess the promise of this app’s approach for patients who are acutely suicidal and inform our and others’ development of digital innovations used for behavioral and medical interventions, including telehealth delivery. We predicted that, compared with care as usual (CAU) control participants, Jaspr Health participants would receive more evidence-based suicide prevention interventions, report greater reductions in their agitation and distress, indicate superior capacity for coping with their suicidal ideation over time, and exhibit higher patient satisfaction with their overall ED experience.

## Methods

### Design and Recruitment

We recruited individuals who were acutely suicidal and seeking ED-based psychiatric crisis services from 2 EDs located in the Midwest. Although a number of large health care systems in geographically diverse regions of the United States had intended to participate in the research, only 2 systems had completed the necessary contractual procedures at the start of the study; efforts to complete contracting with other systems ceased with COVID-19. Both health care systems identified their participating EDs. In both cases, EDs were selected on the basis of serving a large number of patients who are suicidal and the ED staff’s willingness to incorporate the research into their workflow. Each ED offered 24/7 psychiatric care offered by behavioral health providers. One site used master’s level social workers to perform an initial clinical assessment and recommended discharge disposition for later review by an ED physician. The other site had a psychiatrist and psychiatric nurse practitioner embedded in the ED to perform the initial suicide assessment and clinical intervention, with the psychiatrist determining the discharge disposition. All study procedures took place in the patient’s ED room.

An advisory group of people with lived experience (PLE) with suicide assisted in developing research procedures to ensure the acceptability of the research method. Before finalizing the research protocol (measures, scripts, and methods), a timed ED protocol simulation test was conducted individually with 4 PLE advisors that fully mirrored the ED research experience. Advisors provided their impressions on a number of issues such as the overall length and relevance of the research measures, including whether they were acceptable given the cognitive load experienced by suicidal persons while in the ED and ensuring the researcher script was clear, easy to understand, and in plain speech (rather than scientific or clinical language that may be confusing or off-putting). Concerns expressed by one PLE were treated as hypotheses to verify with another. By applying a user-centered design method commonly used when developing software, modifications were made until the protocol was deemed acceptable (content, process, and time required to complete) by investigators, the Director of Lived Experience Integration, and PLE advisors. All procedures were approved by a full board review by the Sterling Institutional Review Board and the Institutional Review Board at the Catholic University of America. External monitoring was provided by an independent Data Safety Monitoring Board comprising recognized suicide experts. The trial was registered at ClinicalTrials.gov NCT03584386.

Eligible participants were English-speaking adults, 18 years or older, and acutely suicidal in the ED. Patients who were actively psychotic, severely agitated, and/or significantly impaired by alcohol or drugs were excluded from participating because they would be unable to provide informed consent and participate meaningfully in a behavioral intervention and/or because of safety concerns involving access to a tablet that could be weaponized.

Potentially eligible participants were identified by a member of the medical team or behavioral health specialists who initially approached each patient to briefly describe the study and assess their interest in participating. A researcher then met with the patient, provided a high-level summary of the project to determine the patient’s interest, and conducted an eligibility screen to verify that the patient met the study criteria. As patients who are suicidal are a vulnerable population, great care was taken to ensure that before providing consent, eligible participants had a thorough understanding of the study procedure, including its risks and benefits. To standardize the informed consent process and ensure that all information was reliably, simply, and succinctly delivered, all eligible participants watched a brief 5-min video in which the study’s Director of Lived Experience Integration walked through all study procedures aided by a simple PowerPoint illustrating the key points. Eligible participants were offered the opportunity to review the written informed consent form and/or to have the researcher review other specific sections. To minimize enrollment bias, randomization to either Jaspr Health or the CAU condition occurred after the process of informed consent was performed. A minimization randomization procedure was used to match participants to condition based on suicide severity and earlier history of ED visits for suicidal behavior. To guard against bias or possible disappointment caused by not being randomly assigned to the Jaspr Health condition, specific details about Jaspr Health were contained in a separate supplemental 2-min video, and consent was provided to those assigned to Jaspr Health following the randomization procedure.

Following the completion of the informed consent and randomization process, participants completed a baseline assessment using a tablet. In the CAU condition, participants completed the posttest assessment 2 hours after the baseline assessment. In the Jaspr Health condition, participants were given up to 2 hours to use the app and then administered the posttest assessment. The 2-hour study time and app use were paused when Jaspr Health participants met with a member of their care team and then resumed when done. To ensure safety while using the tablet-based app, the researcher remained in the patient’s room during their use and sat on a chair in the corner of the room. Researchers told patients that they would be focusing on their own work to minimize the impact of their presence. Researchers were allowed to answer specific questions asked by the patient about Jaspr Health use but did not speak to the patient during the study session. Jaspr Health participants also received access to usual care.

### Measurements

Study data were collected using SurveyMonkey, a Health Insurance Portability and Accountability Act of 1996 (HIPAA)–compliant secure web-based assessment tool and were stored in a HIPAA-compliant cloud-based server. Participants completed self-report questionnaires on an Apple iPad tablet. Researchers entered additional data (eg, time spent using Jaspr Health and answers to semistructured interview questions) onto laptops where content was saved and stored on the cloud-based server.

Key domains assessed among those in the Jaspr Health group included feasibility and acceptability. Feasibility was measured by the absence of negative or adverse events, the premature *stopping* of a test session by medical personnel concerned about the patient’s welfare, or the premature disengagement of use (requesting to stop after 20 min, the average length of an ED-based clinical interview). Acceptability was measured by the total number of minutes used, whether the patient would recommend Jaspr Health to others in their situation, and satisfaction.

RCT measures were developed in collaboration with the Emergency Department Safety Assessment and Follow-up Evaluation [42] principal investigator and Jaspr Health consultant (Dr Boudreaux), reviewed with PLEs, and selected for their brevity and simplicity for use with individuals who are suicidal and seeking psychiatric crisis services in an ED. The *Safety and Imminent Distress Questionnaire* is a 4-item, face-valid self-report survey based on Dr Boudreaux’s Keeping Myself Safe Subject Usability Survey [43]. Participants rated their feelings in the present moment using a 10-point scale. The following items were included: intensity of emotional distress (1=no distress; 10=highest distress ever felt), the extent to which they felt calm or agitated (1=very calm; 10=very frustrated or agitated), their ability to cope with thoughts of killing themselves (1=no ability to cope; 10=strong ability to cope), and their ability to go home safely (1=not able; 10=very able). The *Suicide-Related Coping Scale* (SRCS) [44] is a 17-item psychometrically sound self-report measure of coping with suicidal thoughts, urges, and crises. The SRCS uses a 5-point rating scale (0=strongly disagree; 4=strongly agree). The Emergency Room-Patient Satisfaction Survey (ER-PSS) is a 7-item measure used to assess patient experience in the ED. The measure was developed in consultation with the patient experience division of a large reputable health care organization. The initial 6 items used a 5-point rating scale (1=poor; 5=excellent). Items included the helpfulness of ED visit, the degree to which the patient felt listened to and cared about by their care team, the likelihood that they would recommend the ED to others in their situation, and their overall rating of care they received. A final item involves rating their overall ED experience from 1 (worst) to 100 (best). The *Jaspr Health Patient Satisfaction Questionnaire* is an 8-item survey that adapts the ER-PSS to evaluate Jaspr Health, including its ease of use and helpfulness to patients. Patients also rate Jaspr Health on a 100-point scale and indicate whether they would recommend Jaspr Health to others in their situation. A *brief semistructured interview* was conducted at the end of the posttest session and sought to identify what, if any, suicide prevention best practices the participant received while in the ED. When they positively affirmed receiving an intervention, patients were asked to subjectively rate the thoroughness with which they received best practices using a 5-point Likert scale (1=not very thorough; 5=very thorough). They were also asked who delivered the best practice (a member of their care team, Jaspr Health, or both). On average, baseline and posttest measures took less than 10 min to complete, and the semistructured interview took approximately 3 min to administer.

### Intervention

All clinical interventions contained in Jaspr Health draw upon well-established evidence-based practices for suicide prevention that are recommended for adults in the ED who are suicidal [45]. Guided by the CAMS Suicide Status Interview, an adaptation of the Suicide Status Form, Jaspr Health includes an artificial intelligence–powered virtual guide chatbot that gathers patient self-report: the guide *conducts* the comprehensive suicide assessment, *discusses* the importance of lethal means safety management and collaboratively generates a plan to reduce or eliminate access during the high-risk period, and generates a crisis stabilization plan with the patient. Content from these chatbot-driven *discussions* are then summarized in a clinical decision support guide for use by the care team in deriving an evidence-based discharge disposition.

In light of the increased awareness of the power of imparting messages of hope and insights by PLEs [46-50], Jaspr Health also includes psychoeducation videos delivered by PLEs on what to expect in the ED, how to survive the first days after returning home from the hospital, coping with shame, strategies for staying well, and inspiring messages to generate hope (eg, *My Wish for You*). Efficacious behavioral skills from Dialectical Behavior Therapy [51-63], a recognized gold standard in the treatment of suicidal behavior, are also included to teach users how to tolerate distress, change unwanted negative emotions, distract from painful cues, better manage their thoughts with mindfulness, and radically accept that which cannot be changed. If used as intended, Jaspr Health may significantly increase the routine delivery of evidence-based suicide prevention interventions compared with usual care while decreasing potential exposure to malpractice liability through improved suicide-focused practice and extensive documentation.

### Statistical Analysis

We used descriptive statistics, chi-square tests, and generalized linear model (GLM). Descriptive statistics (means and percentages) described the sample, satisfaction with Jaspr Health, and number of evidence-based interventions received. GLM compared Jaspr Health and CAU on satisfaction with their ED experience and the amount of change from baseline to posttest (using GLM’s generalized estimating equations [GEEs]). These GLM analyses controlled for which ED patients were in and for age (which we found differed between conditions). Chi-square tests compared conditions on what intervention they received. Power was low for comparing means and proportions between conditions, with a very large effect size (Cohen *d*=1.05) needed to detect a statistically significant (*P*=.048) difference (power of 0.80, two-tailed test). Power was higher for detecting a statistically significant time×condition effect in GEE analysis, with the ability to detect a medium effect size (*f*=0.27).

## Results

### Enrollment and Participant Characteristics

**Figure 1:**
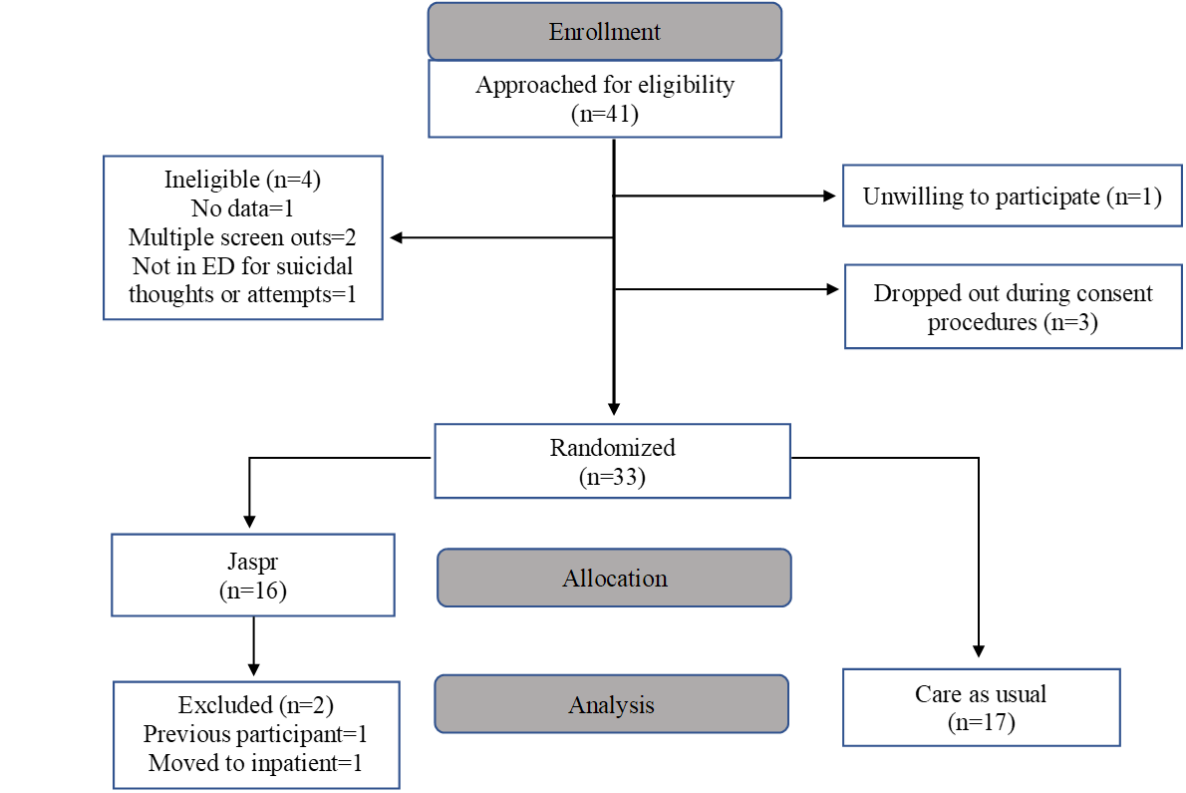
Participant enrollment CONSORT (Consolidated Standards of Reporting Trials) flow chart. ED: emergency department.

For approximately 2 months (January through February 2020), 41 patients who were suicidal in the ED were approached, screened, and informed of the study. Of these, 33 consented to being randomized to Jaspr Health (n=16) or CAU (n=17). Two Jaspr Health participants were excluded from participation after randomization (one was transferred to an inpatient unit before beginning the intervention and the other had previously participated in an earlier usability study), resulting in a Jaspr Health sample size of 14. Unfortunately, the rapid spread of COVID-19 required the suspension of recruitment efforts at participating health care organizations, thus ending this phase of research earlier than planned and with a significantly smaller sample size than originally planned (N=90). Figure 1 provides a consort flowchart of the enrollment.

Of the sample, 65% (20/31) were identified as female and 87% as White (27/35). Participants’ ages ranged from 18 to 68 years, and the average age was 34.4 years (SD 15.17). A total of 32% (10/31) of the sample graduated from high school, 23% (7/31) obtained a 2-year college degree, 13% (4/31) earned a 4-year college degree, and 10% (3/31) had earned a graduate degree. Of the 31 participants, 25 (82%) had made a suicide attempt in their lifetime and 16 (64%) had made 2 or more attempts in their lifetime. In addition, 61% (19/31) of participants reported a lifetime history of engaging in nonsuicidal self-injurious behaviors and at an average rate of 8.8 times (median=2.0) during the past 3 months. Moreover, 55% (17/31) of participants indicated that they had visited the ED for suicidal behaviors 3 or more times in their lifetime; of this subsample, 77% (23/31) of participants had been to the ED before the index ED visit 1 to 2 times in the past 3 months for suicidal behaviors. Overall, 48% (15/31) of participants had sought psychiatric crisis services in the ED on 3 to 7 occasions in their lifetime. Participants had already been in the ED for an average of 17 hours before enrolling in the research study. With the exception of age, no differences were detected between conditions at baseline on demographics and baseline measures. Furthermore, no differences between sites were detected for gender, race, education, use of emergency services, or suicidal-related variables (eg, suicide severity and history of attempts and nonsuicidal self-injury). Table 1 shows participants’ characteristics by condition.

**Table 1:** Participants’ characteristics by study group (enrollment).

Participant characteristic^a^			Jaspr Health (n=14)		CAU^b^ (n=17)
Age (years), mean (range, SD)			29 (19-49, 10.76)		39 (18-68, 16.92)
**Gender,^c^** **n (%)**					
	Female	8 (57)		12 (71)	
	Male	6 (43)		5 (29)	
**Race,^d^** **n (%)**					
	Black or African American	0 (0)		1 (6)	
	White	12 (86)		15 (88)	
	More than one race or other	2 (14)		1 (6)	
**Education, n (%)**					
	Less than high school	1 (7)		1 (6)	
	High school graduate	3 (21)		7 (41)	
	Some college	3 (21)		2 (12)	
	2-year college degree or trade school	4 (29)		3 (18)	
	4-year college degree	2 (14)		2 (12)	
	4-year degree+masters	1 (7)		2 (12)	
**Suicide severity and history, n (%)**					
	No attempt	3 (21)		3 (18)	
	1 attempt	5 (36)		4 (24)	
	2 or more attempts	6 (43)		10 (59)	
**Emergency services’ use history, n (%)**					
	No previous ED^e^ visits	2 (14)		3 (18)	
	1 or 2 ED visits	6 (43)		3 (18)	
	3 or more ED visits	6 (43)		11 (64)	
**Emergency services used in the past 3 months, n (%)**					
	1-2 times	11 (79)		13 (77)	
	3-4 times	2 (14)		1 (6)	
	5-7 times	0 (0)		0 (0)	
	7-10 times	1 (7)		1 (6)	
	More than 10 times	0 (0)		1 (6)	
**Emergency services used across lifespan, n (%)**					
	1-2 times	7 (50)		4 (24)	
	3-4 times	5 (36)		5 (29)	
	5-7 times	2 (14)		3 (18)	
	7-10 times	0 (0)		2 (12)	
	More than 10 times	0 (0)		2 (12)	
**Nonsuicidal self-injury, n (%)**					
	Yes	7 (50)		12 (71)	
	No	7 (50)		5 (29)	

^a^Numbers and percentages may not sum to total because of missing data.

^b^CAU: care as usual.

^c^One sex assignment was different from birth.

^d^One participant identified as Hispanic.

^e^ED: emergency department.

### Feasibility and Acceptability of Digital Technology

Factors representative of the feasibility and acceptability of Jaspr Health showed strong, positive results. All Jaspr Health participants completed the use of digital technology without any adverse events or premature stopping of the test session either by medical personnel or participants. Jaspr Health participants used Jaspr Health for an average of 80 min (SD 33 min; median 85 min). All Jaspr Health participants indicated that they would recommend the digital tool to other suicidal individuals in their situation. In addition, participants gave Jaspr Health high satisfaction ratings. As shown in Table 2, the average satisfaction rating for Jaspr Health was 4.4 (SD 0.63) using a 5-point Likert scale, where 1=poor and 5=excellent.

**Table 2:** Satisfaction ratings among Jaspr Health participants^a^.

Item	Mean (SD)
Jaspr was easy to use and understand	4.5 (0.76)
Helpfulness or Jaspr	4.1 (0.77)
Felt cared about Jaspr	3.9 (0.92)
Helpfulness of information	4.1 (1.00)
Overall rating of care by Jaspr	4.4 (0.63)

^a^Response categories for Jaspr satisfaction items coded from 1 to 5: 1=poor; 2=fair, 3=good, 4=very good, and 5=excellent.

### Feasibility and Effectiveness

Key findings favored the Jaspr Health over the CAU condition. As shown in Table 3, Jaspr Health participants reported receiving significantly more of the best practice interventions recommended for suicidal individuals while in the ED. In addition, Jaspr Health participants indicated robust exposure to these interventions. As shown in Table 4, they reported learning an average of 3 new behavioral skills (SD 1.3) and *engaged* with 4 PLEs (SD 2.63). The degree of thoroughness with which Jaspr Health participants received best practices ranged from an average of 3.4 (SD 1.1; crisis plan) to 4.1 (SD 0.86; PLE) on the 5-point scale.

**Table 3:** Participants responding yes to receiving interventions.

Variable	CAU^a^ (n=17), n (%)	Jaspr (n=14), n (%)	*χ*^2^ (*df*)	*P* value
Crisis stabilization plan	2 (12)	14 (100)	23.9 (1)	<.001
Lethal means counseling	1 (6)	12 (85)	19.0 (1)	<.001
Skills	2 (12)	13 (93)	20.2 (1)	<.001
PLE^b^	1 (6)	13 (93)	23.4 (1)	<.001

^a^CAU: care as usual.

^b^PLE: people with lived experience with suicide.

**Table 4:** Interventions received across conditions: number received and rating of thoroughness (n=31).

Intervention	Number received, mean (SD)	Thoroughness^a^, mean (SD)
Crisis stabilization plan	—^b^	3.4 (1.08)
Lethal means counseling	—^b^	3.5 (1.31)
Skills	2.7 (1.30)	3.7 (1.38)
PLE^c^	3.7 (2.63)	4.1 (0.86)

^a^Response categories for thoroughness coded from 1 to 5: 1=not very thorough and 5=very thorough.

^b^Participants did not receive a quantifiable number of interventions for crisis planning or lethal means counseling.

^c^PLE: people with lived experience.

Table 5 shows that Jaspr Health participants had greater improvement than CAU participants from baseline to posttest in suicide-related coping, capacity to cope with distress, and agitation and distress using GEE analysis. Statistically significant time×condition effects show that during the 2-hour experimental procedure, compared with CAU patients, Jaspr Health patients reported greater decreases in intensity of agitation and distress and greater increases in their ability to cope with thoughts of killing themselves. Within-condition effect sizes were large to very large for Jaspr Health participants’ decreases in agitation and distress (Cohen *d*=0.61 and 1.00, respectively) and increases in coping ability (Cohen *d*=0.90). In contrast, effect sizes for CAU participants were small. Specifically, a decrease in distress (Cohen *d*=0.33), a small increase in agitation (*d*=0.11), and an increase in coping ability (Cohen *d*=0.32) were observed in CAU. Although the time×condition effect for readiness to go home safely was not statistically significant, effects sizes were small for CAU but larger (though still small in magnitude) for Jaspr Health. Finally, compared with CAU, Jaspr Health participants reported a significant time×condition effect, reflecting a greater increase in their SRCS-measured suicide-related coping capability than CAU participants, with a very large effect size for Jaspr Health (Cohen *d*=1.11) compared with a small effect for CAU (Cohen *d*=0.26).

**Table 5:** Repeated measures analysis comparing Jaspr and care as usual on pre- and postintervention outcomes.

Scales or items		Baseline, mean (SD)	Postintervention, mean (SD)	Time				Time×condition				Cohen *d*^a^: within-condition change	
				*χ*^2^ (*df*)		*P* value		*χ*^2^ (*df*)		*P* value			
**SRCS^b^**					17.3 (1)		<.001		8.1 (1)		.004		
	Jaspr^c^	34.8^d^ (11.00)	44.8 (11.69)									1.11	
	CAU^e,f^	37.6 (13.28)	39.5 (14.16)									0.26	
**SIDQ^g^** **—Distress**					15.8 (1)		<.001		5.5 (1)		.02		
	Jaspr	6.7^h^ (2.02)	4.4 (2.38)									1.00	
	CAU	7.3 (2.71)	6.7 (2.49)									0.33	
**SIDQ—Agitation**					3.6 (1)		.06		5.5 (1)		.02		
	Jaspr	5.9^i^ (2.61)	4.23 (2.24)									0.61	
	CAU	6.1 (2.76)	6.3 (2.14)									0.11	
**SIDQ—Coping ability**					13.2 (1)		<.001		5.8 (1)		.02		
	Jaspr	4.6^j^ (2.28)	6.6 (2.71)									0.90	
	CAU	4.8 (2.41)	5.2 (2.28)									0.32	
**SIDQ—Readiness to go home**					1.2 (1)		.27		0.5 (1)		.49		
	Jaspr	7.0^k^ (3.32)	7.8 (2.26)									0.28	
	CAU	4.0 (3.33)	4.4 (2.95)									0.05	

^a^Interpretation of Cohen *d* is 0.20 small, 0.50 medium, and 0.80 large.

^b^SRCS: Suicide-Related Coping Scale.

^c^Analysis sample size of Jaspr, n=14.

^d^Response categories for suicide-related coping coded from 0 to 4: 0=strongly disagree and 4=strongly agree.

^e^Analysis sample size of care as usual, n=17.

^f^CAU: care as usual.

^g^SIDQ: Safety and Imminent Distress Questionnaire.

^h^Response categories, for distress coded from 1 to 10: 1=no distress and 10=highest distress ever felt.

^i^Response categories for agitation coded from 1 to 10: 1=very calm and 10=very frustrated or agitated.

^j^Response categories, for coping ability coded from 1 to 10: 1=no ability to cope and 10=strong ability to cope.

^k^Response categories, for readiness to go home coded from 1 to 10: 1=not able and 10=very able.

Although not generally statistically significant, Table 6 shows that effect sizes comparing Jaspr Health and CAU conditions on ED patient satisfaction favored Jaspr Health and ranged from medium to large in magnitude. A large effect size (Cohen *d*=0.80) and a nearly statistically significant difference (*P*=.06) was observed on arguably the most important ED patient satisfaction item *Overall Rating of Care*, again favoring Jaspr Health.

**Table 6:** *t* tests comparing Jaspr and care as usual on emergency department satisfaction measures.

Items^a^	Jaspr (n=14), mean (SD)	CAU^b^ (n=17), mean (SD)	Between condition		
			*χ*^2^ (*df*)	*P* value	Cohen *d*^c^
Helpfulness of ER^d^ visit	3.8 (0.98)	3.2 (1.19)	2.2 (1)	.14	0.56
Felt listened to	4.2 (0.98)	3.5 (1.23)	2.0 (1)	.15	0.63
Felt cared about	4.1 (1.23)	3.7 (1.12)	1.1 (1)	.29	0.42
Ready to return home	3.4 (1.50)	2.8 (1.44)	0.6 (1)	.43	0.41
Recommend ER	4.3 (1.20)	3.5 (1.23)	2.6 (1)	.11	0.65
Overall rating of care	4.2 (0.98)	3.4 (1.17)	3.7 (1)	.06	0.80

^a^Response categories for ED satisfaction items coded from 1 to 5: 1=poor, 2=fair, 3=good, 4=very good, and 5=excellent.

^b^CAU: care as usual.

^c^Interpretation of Cohen *d* is 0.20 small, 0.50 medium, and 0.80 large.

^d^ER: emergency room.

## Discussion

### Relevance and Findings

We wondered whether patients who are acutely suicidal would tolerate interacting with an artificial intelligence–powered chatbot designed to deliver evidence-based suicide-focused interventions. Would they also choose to virtually hear from PLE and learn behavioral skills to increase their capacity to cope with distress? If they did, would it produce positive clinical outcomes and improve their overall ED visit experience?

Preliminary findings strongly supported Jaspr Health’s feasibility and acceptability, while also appearing promising as an effective clinical intervention. With respect to feasibility and acceptability, patients who are suicidal in the ED tolerated Jaspr Health and opted to use the app on their own for a median of 85 min. Of 14 Jaspr Health patients, all completed a comprehensive suicide assessment and created a crisis stabilization plan, and 12 (85%) patients engaged in lethal means counseling. Jaspr Health participants also opted to learn 3 behavioral skills and hear from 4 PLEs and gave Jaspr Health high satisfaction ratings—100% recommended it for use by others in their situation. In addition, no adverse events occurred during its use. Jaspr Health appeared clinically effective. In comparison with CAU participants, those receiving Jaspr Health reported statistically significant reductions in agitation and distress over time and improved capacity to cope with current and future suicidal thoughts. They also felt more positively about their overall ED experience. These findings are not surprising, given that those who received Jaspr Health received the evidence-based interventions recommended by experts at a much higher and statistically significant rate compared with CAU patients. Only 12% (2/17) of CAU patients had developed a crisis stabilization plan while in the ED, and only 6% (1/17) of CAU patients discussed the lethal means with his or her care team. The findings are particularly noteworthy, given that the study was underpowered because of the sudden need to stop the study because of COVID-19.

Although still in the early stages, the implications of this study are substantial. First, this study demonstrates that digital technologies can be used to fulfill the mandate and vision of aiding the delivery of evidence-based suicide prevention care to patients who are acutely suicidal while in the ED. Powerful interventions supported by decades of suicide prevention clinical research can reach *and impact* those they are designed to help without significant demands on personnel or extensive training. Indeed, care teams may actually *save* time, as tools such as Jaspr Health reliably and compassionately gather relevant information from the patient that can be used by the provider to deepen their own clinical interview. Second, delivering state-of-the-art evidence-based care for people who are in an acute suicide crisis can be performed anytime and anywhere there is internet access and a tablet computer, including rural and frontier communities with unusually high rates of suicide and limited access to psychiatric care. By extension, digital technologies such as Jaspr Health may be blended with other crisis stabilization services and delivered via telehealth, which reduces the need for some to go to the ED altogether. They can also be used to support the standardized delivery of evidence-based care to patients who are suicidal or admitted to a medical or surgery unit for injuries resulting from a suicide attempt or in a primary care context. When integrated into a health care system’s electronic health record, such tools may augment (not replace) a trained medical personnel’s interventions and improve the overall quality of care. They may also help mitigate malpractice claims by ensuring thorough documentation of specific evidence-based care received by a patient, including ongoing assessment of their suicidality while in the ED [64].

### Limitations

This preliminary study is the first of its kind that we are aware of where digital technology was used to intervene with a highly vulnerable suicidal population seeking psychiatric crisis services in an ED. The study contained a number of inherent methodological limitations, as a feasibility- and acceptability-oriented RCT. Although developmentally appropriate for a study at this early phase, the threats to internal validity are notable. First, researchers were only in the room for Jaspr Health, but not CAU, to ensure safe use of the app during initial testing. Although the researchers were instructed to not engage in conversation with the participants (in fact, they were instructed to appear busy on their laptops), their presence alone may have been a factor that accounted for a reduction in distress and agitation compared with CAU. Furthermore, their presence may have also positively affected study outcomes via a social desirability bias. Second, a placebo device was not used for the CAU participants. Without controlling for the tablet itself and engagement with it, we cannot know for certain whether the effect achieved was because of Jaspr Health’s content or simply the outcome of having access to a tablet. (It is worth noting, however, that all rooms were equipped with a television for patient use). Third, the assessors were not blinded to the patients’ study condition. Finally, limitations of budget and project scope, complicated further by reducing study length because of COVID-19, resulted in our inability to expand to other ED research sites located in more ethnically and racially diverse regions of the United States. This resulted in another significant study limitation, namely, a predominantly White sample that significantly limits the study’s external validity. Future research should focus specifically on ED sites in ethnically and racially diverse regions of the United States to ensure greater sample diversity to address this considerable limitation.

### Conclusions

This pilot study provides preliminary support for an approach that may reduce suicide by delivering powerful evidence-based suicide prevention assessments and interventions for suicidal ED patients at particularly high risk for death by suicide. It also paves the way for digital innovations to solve complex behavioral health problems by improving reliable delivery of evidence-based practices, enhancing patient experiences, and producing compelling clinical outcomes while aiding and not taxing busy care providers. It also highlights the need to accelerate efforts to improve the delivery of evidence-based suicide prevention practices in EDs. Despite the study’s limitations, which include threats to internal validity in the RCT and the small sample size, the use of digital technologies appears feasible and acceptable to both patients *and* their care teams, even for a highly vulnerable population in a complex and fast-paced environment. In addition to a statistically significant reduction in distress and agitation compared with CAU and increased capacity to cope with current and future suicidal thoughts, perhaps the most notable finding is that those using the digital solution actually *received* evidence-based suicide-focused interventions. Digital solutions such as Jaspr Health also allow hospital-based care teams to improve their own clinical impact by using a chatbot to gather important information that can then be used in subsequent clinical discussions with the patient. Future studies should seek to reduce threats to internal validity by building in greater experimental controls while also recruiting participants from more ethnically and racially diverse regions of the country to extend opportunities for ethnic and racial minorities to participate and increase external validity, thereby saving the lives of people in need.

## Appendix

Multimedia Appendix 1 CONSORT-eHEALTH checklist (V 1.6.1).

## Abbreviations

CAMS: Collaborative Assessment and Management of Suicidality
CAU: care as usual
ED: emergency department
ER-PSS: Emergency Room-Patient Satisfaction Survey
GLM: generalized linear model
GEE: generalized estimating equations
HIPAA: Health Insurance Portability and Accountability Act of 1996
PLE: people with lived experience
RCT: randomized controlled trial
SRCS: Suicide-Related Coping Scale
